# Clinical course and outcome of 107 patients infected with the novel coronavirus, SARS-CoV-2, discharged from two hospitals in Wuhan, China

**DOI:** 10.1186/s13054-020-02895-6

**Published:** 2020-04-30

**Authors:** Dawei Wang, Yimei Yin, Chang Hu, Xing Liu, Xingguo Zhang, Shuliang Zhou, Mingzhi Jian, Haibo Xu, John Prowle, Bo Hu, Yirong Li, Zhiyong Peng

**Affiliations:** 1 Department of Critical Care Medicine, Zhongnan Hospital of Wuhan University, Wuhan, 430071 Hubei China; 2 Department of Ultrasound Medicine, Zhongnan Hospital of Wuhan University, Wuhan, 430071 Hubei China; 3 Department of Critical Care Medicine, Xishui Hospital, Huanggang, Hubei China; 4 Department of Health Education, Center for Disease Control and Prevention, Shaoxing, 312000 Zhejiang China; 5 Department of Radiology, Zhongnan Hospital of Wuhan University, Wuhan, 430071 Hubei China; 6 Adult Critical Care Unit, The Royal London Hospital, Barts Health NHS Trust, London, UK; 7 Department of Laboratory Medicine, Zhongnan Hospital of Wuhan University, Wuhan, 430071 Hubei China

**Keywords:** Coronavirus, Infection, Pneumonia

## Abstract

**Background:**

In December 2019, coronavirus disease 2019 (COVID-19) outbreak was reported from Wuhan, China. Information on the clinical course and prognosis of COVID-19 was not thoroughly described. We described the clinical courses and prognosis in COVID-19 patients.

**Methods:**

Retrospective case series of COVID-19 patients from Zhongnan Hospital of Wuhan University in Wuhan and Xishui Hospital, Hubei Province, China, up to February 10, 2020. Epidemiological, demographic, and clinical data were collected. The clinical course of survivors and non-survivors were compared. Risk factors for death were analyzed.

**Results:**

A total of 107 discharged patients with COVID-19 were enrolled. The clinical course of COVID-19 presented as a tri-phasic pattern. Week 1 after illness onset was characterized by fever, cough, dyspnea, lymphopenia, and radiological multi-lobar pulmonary infiltrates. In severe cases, thrombocytopenia, acute kidney injury, acute myocardial injury, and adult respiratory distress syndrome were observed. During week 2, in mild cases, fever, cough, and systemic symptoms began to resolve and platelet count rose to normal range, but lymphopenia persisted. In severe cases, leukocytosis, neutrophilia, and deteriorating multi-organ dysfunction were dominant. By week 3, mild cases had clinically resolved except for lymphopenia. However, severe cases showed persistent lymphopenia, severe acute respiratory dyspnea syndrome, refractory shock, anuric acute kidney injury, coagulopathy, thrombocytopenia, and death. Older age and male sex were independent risk factors for poor outcome of the illness.

**Conclusions:**

A period of 7–13 days after illness onset is the critical stage in the COVID-19 course. Age and male gender were independent risk factors for death of COVID-19.

## Background

In late 2019, a novel coronavirus, designated severe acute respiratory syndrome coronavirus 2 (SARS-CoV-2), was identified as the cause of COVID-19 in Wuhan, a city in the Hubei province of China [1, 2]. Full-genome sequencing and phylogenic analysis indicated that SARS-CoV-2 is a betacoronavirus in the same subgenus as the SARS virus, but in a different clade [2]. SARS-CoV-2 is 96% identical at the whole-genome level to a bat coronavirus, suggesting that bats are the primary source [3, 4]. Epidemiologic investigations of initial cases showed COVID-19 was linked with exposure to the Wuhan seafood market which also sold live rabbits, snakes, and other animals [5]. Subsequently, human-to-human transmission among close contacts has been the primary mechanism of transmission [6]. The disease has spread rapidly around the world, and more than 410,000 cases of COVID-19 have been reported. COVID-19 outbreak has been reported in other countries, mainly among travelers from Wuhan and their contacts [7, 8]. WHO has declared this disease a pandemic.

The incubation period of COVID-19 is thought to be up to14 days following exposure [5, 6, 9]. The principal presenting features of COVID-19 are fever, cough, dyspnea, and bilateral infiltrates on chest imaging [10, 11]. Approximately 20% of patients progress to multi-organ dysfunction (including respiratory failure, septic shock, acute cardiac injury, or acute renal failure [10–12]. However, a complete picture of the clinical course of COVID-19 has not been described thoroughly [13]. Except for infection control and supportive therapy, there is no specific therapy of COVID-19. Multiple organ support therapy is the cornerstone in the treatment of critically ill patients with COVID-19 [12, 13]. Early recognition of risk factors for death would be useful to identify those potentially needing critical care at an early stage. Accordingly, a study was conducted to track the clinical course along the entire disease course. A risk factor analysis was performed to reveal important clinical features associated with poor outcomes.

## Methods

### Study design and participants

This case series was approved by the institutional ethics board of Zhongnan Hospital of Wuhan University and Xishui People’s Hospital (No. 2020020). All the discharged (alive at home and dead) patients with confirmed COVID-19 from Zhongnan Hospital of Wuhan University and Xishui People’s Hospital up to February 10, 2020, were enrolled. Oral consent was obtained from patients or patients’ relatives. Zhongnan Hospital, located in Wuhan, Hubei Province, the endemic areas of COVID-19, is one of the major tertiary teaching hospitals and responsible for the treatments for COVID-19 assigned by the government. Xishui People’s Hospital is located in Huanggang city, another early endemic center of COVID-19 in Hubei province. In total, about 340 heath care workers provided care to COVID-19 patients in the two medical centers from January to February 2020. All patients with COVID-19 enrolled in this study were diagnosed according to World Health Organization interim guidance [14]. The methodology of RT-PCR used has been previously reported [12]. The time frame was overlapped with the JAMA cohort, and 88 patients in the current report have been included in the JAMA cohort [12].

### Data collection

The medical records of patients were analyzed by the research team of the Department of Critical Care Medicine, Zhongnan Hospital of Wuhan University. Epidemiological, clinical, laboratory, and radiological characteristics and treatment and outcomes data were obtained with data collection forms from electronic medical records and reviewed by a trained team of physicians. The information recorded included demographic data, medical history, exposure history, underlying comorbidities, symptoms, signs, laboratory findings, chest computed tomographic (CT) scans, treatment measures (i.e., antiviral therapy, corticosteroid therapy, respiratory support, kidney replacement therapy), and outcomes. The date of disease onset was defined as the day when the first symptom was noticed. Acute respiratory distress syndrome (ARDS) was defined according to the Berlin definition [15]. Acute kidney injury (AKI) was identified according to the Kidney Disease: Improving Global Outcomes definition [16]. Cardiac injury was defined if the serum levels of cardiac biomarkers (e.g., troponin I) were above the 99th percentile of the upper reference limit or if new abnormalities were shown in echocardiography. Times from onset of disease to hospital admission, dyspnea, ARDS, ICU admission, and hospital discharge were recorded.

### Statistical analysis

Categorical variables were described using frequencies and percentages, while continuous variables were described using mean, median, and interquartile range (IQR) values. Means for continuous variables were compared using independent group Student’s *t* tests when the data were normally distributed and the Mann-Whitney test when they were not. Proportions for categorical variables were compared using the *χ*^2^ test, although Fisher’s exact test was used when the data were sparse. Univariate analyses were performed to evaluate the risk factors associated with death. Multiple logistic regression analysis was used to identify independent predictors of mortality. All the tests were two-tailed, and *P* value less than 0.05 was considered statistically significant. All analyses were processed by SPSS for Windows version 17.0 (SPSS, Chicago, IL, USA).

## Results

### Basic characteristics

As of February 10, 2020, 544 patients were admitted to Zhongnan Hospital and Xishui Hospital, and 107 patients were discharged. The basic characteristics of the 107 patients (95 from Zhongnan and 12 from Xishui) are shown in Table 1. There were 88 survivors and 19 non-survivors. The median age was 51 years (IQR, 36–65; range, 19–92 years); 57 (53.3%) were male. The median times from first symptoms to hospital admission, dyspnea, and ARDS were 7 days (IQR, 3.5–9), 5.5 days (IQR, 2–9.3), and 7.5 days (IQR, 4.3–11), respectively. The median length of hospital stay was 11 days (IQR, 7–15). In this cohort of 107 patients, hypertension (26 [24.3%]), cardiovascular disease (13 [12.1%]), and diabetes (11 [10.3%]) were the most common coexisting conditions. The most common symptoms at onset of illness were fever (104 [97.2%]), dry cough (67 [62.6%]), fatigue (69 [64.5%]), dyspnea (35 [32.7%]), anorexia (33 [30.8%]), and myalgia (33 [30.8%]). Less common symptoms were sore throat, headache, dizziness, abdominal pain, diarrhea, nausea, and vomiting. At hospital admission, the median respiratory rate was 20/min [IQR, 19–21], and the mean arterial pressure was 89 mmHg [IQR, 83–98].

**Table 1:** Baseline characteristics of COVID-19 patients

Characteristics	Total (*n* = 107)	Survivors (*n* = 88)	Non-survivors (*n* = 19)	*P* value
Age, years	51.0 (36.0–65.0)	44.5 (35.0–58.8)	73.0 (64.0–81.0)	< 0.001*
< 45	46 (43.0)	44 (50.0)	2 (10.5)	0.002
45–59	25 (23.4)	24 (27.3)	1 (5.3)	0.041
60–75	23 (21.5)	16 (18.2)	7 (36.8)	0.119
> 75	13 (12.1)	4 (4.5)	9 (47.4)	< 0.001
Sex				0.003*
Male	57 (53.3)	41 (46.6)	16 (84.2)	
Female	50 (46.7)	47 (53.4)	3 (15.8)	
Comorbidity				
Any comorbidity*	41 (38.3)	28 (31.8)	13 (68.4)	0.003
Hypertension	26 (24.3)	16 (18.2)	10 (52.6)	0.001*
Cardiovascular disease	13 (12.1)	6 (6.8)	7 (36.8)	0.002*
Diabetes	11 (10.3)	6 (6.8)	5 (26.3)	0.024*
Chronic liver disease	6 (5.6)	5 (5.7)	1 (5.3)	1.000
Cerebrovascular disease	6 (5.6)	3 (3.4)	3 (15.8)	0.068
COPD	3 (2.8)	2 (2.3)	1 (5.3)	0.447
Chronic kidney disease	3 (2.8)	2 (2.3)	1 (5.3)	0.447
Symptoms and signs				
Fever	104 (97.2)	85 (96.6)	19 (100.0)	1.000
Dry cough	67 (62.6)	56 (63.6)	11 (57.9)	0.639
Fatigue	69 (64.5)	55 (62.5)	14 (73.7)	0.356
Dyspnea	35 (32.7)	20 (22.7)	15 (78.9)	< 0.001*
Anorexia	33 (30.8)	25 (28.4)	8 (42.1)	0.241
Myalgia	33 (30.8)	28 (31.8)	5 (26.3)	0.638
Pharyngalgia	12 (11.2)	11 (12.5)	1 (5.3)	0.689
Headache	7 (6.5)	7 (8.0)	0 (0)	0.348
Dizziness	7 (6.5)	7 (8.0)	0 (0)	0.348
Diarrhea	7 (6.5)	3 (3.4)	4 (21.1)	0.018*
Nausea	6 (5.6)	6 (6.8)	0 (0)	0.588
Vomiting	3 (2.8)	2 (2.3)	1 (5.3)	0.447
Abdominal pain	2 (1.9)	1 (1.1)	1 (5.3)	0.325
Heart rate (bpm)	86 (75–96)	85 (75–96)	90 (78–100)	0.240
Respiratory rate	20 (19–21)	20 (19–21)	22 (20–24)	0.003*
Mean arterial pressure (mmHg)	89 (83–98)	88 (83–96)	95 (89–101)	0.019*
Onset of symptom to admission (days)	7.0 (3.5–9.0)	7.0 (3.0–9.8)	6.0 (4.0–7.0)	0.405
Onset of symptom to dyspnea (days)	5.5 (2.0–9.3)	7.0 (3.3–10.8)	4.0 (1.8–7.5)	0.103
Onset of symptom to ARDS (days)	7.5 (4.3–11.0)	10.0 (6.0–13.0)	7.0 (3.5–9.0)	0.081
Length of hospital stay (days)	11.0 (7.0–15.0)	10.5 (7.0–14.0)	14.0 (6.0–17.0)	0.561

Data are median (IQR), *n* (%). *P* values indicate differences between survivors and non-survivors. *P* < 0.05 was considered significant. Vital signs including heart rate, respiratory rate, and mean arterial pressure were collected at admission

*COPD* chronic obstructive pulmonary disease, *ARDS* acute respiratory distress syndrome, *bpm* beats per minute

*One patient had the comorbidity of lung cancer and died of ARDS

In comparison with the 88 hospital survivors, the 19 non-survivors were significantly older (median age, 73 years [IQR, 64–81] vs 44.5 years [IQR, 35–58.8]; *P* < .001) and were predominantly male (16 [84.2%] vs 41 [46.6%]; *P* = .003). Non-survivors were more likely to have underlying comorbidities, including hypertension (10 [52.6%] vs 16 [18.2%]; *P* = .001) and other cardiovascular diseases (7 [36.8%] vs 6 [6.8%]; *P* = .002). Compared with the survivors, non-survivors were more likely to report dyspnea (15 [78.9%] vs 20 [22.7%]; *P* < .001) and diarrhea (4 [21.1%] vs 3 [3.4%]; *P* = .018) at presentation. At hospital admission, the respiratory rate was higher in survivors than in non-survivors (22 [IQR 20–24] vs 20 [17–19]; *P* = .003). Similarly, the mean arterial pressure was higher in non-survivors than in survivors (95 mmHg [IQR 89–101] vs 88 mmHg [83–96]; *P* = .019).

### Laboratory values and radiographic findings

Laboratory values and radiographic findings at hospital admission are shown in Table 2. Lymphopenia (0.9 × 10^9^/L [0.7–1.2]) and prolonged prothrombin time (12.8 [11.9–13.5]) at admission were prominent features. Ninety (84.1%) patients showed multi-lobar involvement on initial radiographs. One hundred five (98.1%) patients showed bilateral involvement on chest CT scan during hospitalization. Compared with survivors, on admission, non-survivors had higher neutrophil counts (5.4 × 10^9^/L [3.2–8.5] vs 2.8 × 10^9^/L [2–3.9], *P* < 0.001), lower platelet count (122 × 10^9^/L [83–178] vs 178 [139–207], *P* = 0.006), and higher D-dimer level (439 mg/L [202–1991] vs 191 mg/L [108–327], *P* = 0.003). Admission values of blood urea, creatinine, highly sensitive troponin I, serum creatine kinase, creatine kinase-MB, lactate dehydrogenase, alanine aminotransferase, and aspartate aminotransferase were also significantly higher in the non-survivors.

**Table 2:** Laboratory values and radiographic findings at the admission of COVID-19 patients

	Normal range	Total (*n* = 107)	Survivors (*n* = 88)	Non-survivors (*n* = 19)	*P* value
White blood cell count, × 10^9^/L	3.5–9.5	4.6 (3.7–6.1)	4.4 (3.4–5.8)	6.7 (4.6–10.3)	0.004*
Neutrophil count, × 10^9^/L	1.8–6.3	3.1 (2.1–4.7)	2.8 (2.0–3.9)	5.4 (3.2–8.5)	< 0.001*
Lymphocyte count, × 10^9^/L	1.1–3.2	0.9 (0.7–1.2)	0.9 (0.7–1.3)	0.8 (0.5–1.1)	0.121
Platelet count, × 10^9^/L	125–350	175 (129–200)	178 (139–207)	122 (83–178)	0.006*
Prothrombin time, s	9.4–12.5	12.8 (11.9–13.5)	12.9 (12.0–13.5)	12.6 (11.9–13.5)	0.813
Activated partial thromboplastin time, s	25.1–36.5	31.7 (29.4–33.9)	31.7 (29.5–33.5)	32.7 (27.5–37.0)	0.850
D-dimer, mg/L	0–500	203 (121–358)	191 (108–327)	439 (202–1991)	0.003*
Creatine kinase, U/L	< 171	90 (54–138)	86 (53–121)	142 (87–325)	0.022*
Creatine kinase-MB, U/L	< 25	14 (10–18)	13 (9–16)	18 (13–44)	0.008*
Lactate dehydrogenase, U/L	125–243	236 (176–369)	227 (171–329)	456 (254–588)	0.010*
Alanine aminotransferase, U/L	9–50	23 (16–39)	22 (15–34)	47 (22–66)	0.002*
Aspartate aminotransferase, U/L	15–40	31 (24–47)	29 (23–41)	67 (38–90)	< 0.001*
Total bilirubin, mmol/L	5–21	9.8 (8.4–14.1)	9.5 (8.4–12.9)	11.3 (9.4–20.7)	0.069
Blood urea nitrogen, mmol/L	2.8–7.6	4.2 (3.2–5.6)	3.9 (3.0–4.7)	6.1 (4.9–10.5)	< 0.001*
Creatinine, μmol/L	64–104	71 (60–86)	68 (58–83)	87 (71–130)	< 0.001*
Hypersensitive troponin I, > 26.2 pg/mL, no. (%)	< 26.2	6 (5.6)	1 (1.1)	5 (26.3)	0.001*
Multi-lobar involvement on initial radiographs, no. (%)	NA	90 (84.1)	73 (83.0)	17 (89.5)	0.731
Bilateral involvement on radiographs during hospitalization, no. (%)	NA	105 (98.1)	86 (97.7)	19 (100.0)	1.000

Data are median (IQR) or *n* (%). *P* values indicate differences between survivors and non-survivors. *P* < 0.05 was considered significant. Laboratory values and radiographic findings were collected at admission except that the bilateral involvement on radiographs was collected during hospitalization

*MB* muscle and brain type, *NA* not available

### Clinical profile and laboratory findings in COVID-19 patients

**Fig. 1:**
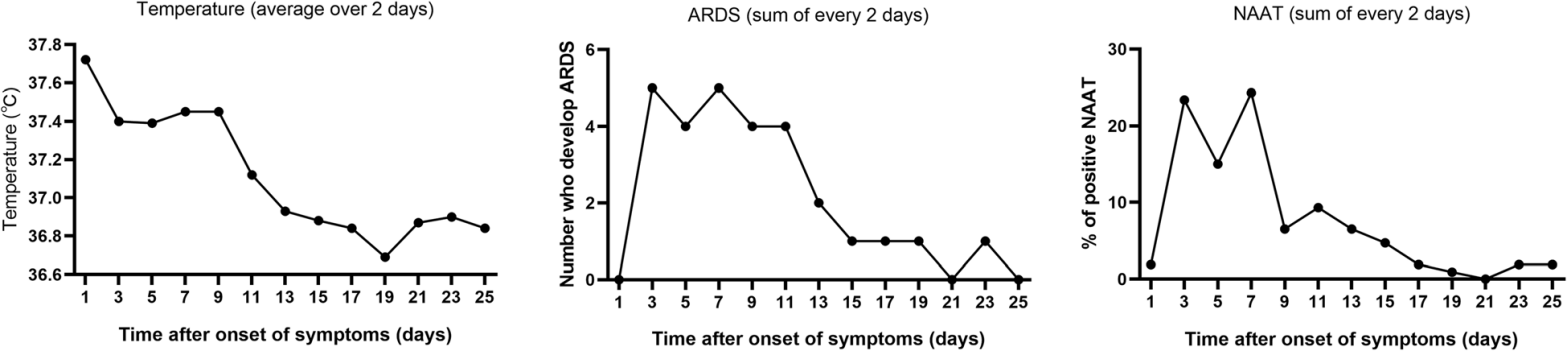
Temporal clinical profiles in 107 patients with COVID-19. % of positive NAAT: percentage of patients who showed positive NAAT for the first time

Temporal clinical profiles in 107 patients with COVID-19 are shown in Fig. 1. Trends of temperature and onset of positive nucleic acid amplification test (NAAT) were consistent. Fever typically lasts for about 10 days. Most patients (about 75%) demonstrated positive NAAT results (measured every 2–3 days) within 9 days after symptom onset. The median time from illness onset to the first positive result of NAAT was 7 days (3.0–10.0), and the duration of active viral shedding was 13 days (IQR, 10–22.3) in survivors. In the majority of cases, the development of ARDS and the need for endotracheal intubation occurred within 9 days after symptom onset.

Dynamic body temperature and laboratory findings in 107 COVID-19 patients are shown in supplementary Fig. 1. During the first week after symptom onset, fever was prominent and more severe in the non-survivors. Body temperature gradually normalized in the second week. In general, white blood cell counts and neutrophil counts were in normal range during week 1, with leukocytosis and neutrophilia as later findings. Lymphopenia was common throughout the disease’s course, and the lymphocyte count dropped more in non-survivors. Platelet counts decreased slightly in the first week, then rose back to normal range rapidly in survivors, but remained low in non-survivors. Mild prolongation of prothrombin time (PT) during the illness course was observed, with no difference between survivors and non-survivors. The D-dimer level was elevated in the non-survivors during the late stage of illness. In the early stage of the illness, higher levels of creatine kinase, creatine kinase-MB, lactate dehydrogenase, alanine aminotransferase, and aspartate aminotransferase were observed in the non-survivors than in the survivors. In non-survivors, blood urea and creatinine levels progressively increased until death.

### Complications, treatments, and outcome

Common complications included ARDS (28 [26.2%]), shock (22 [20.6%]), AKI (14 [13.1%]), and acute cardiac injury (12 [11.2%]) (Table 3). Non-survivors were more likely to have one of these complications than survivors. Secondary infection included 1 case of bacteremia caused by *Staphylococcus caprae* and 4 cases of bacterial pneumonia caused by *Acinetobacter baumannii*. Co-infection with virus included 1 patient tested positive for influenza A, two for influenza B, three for respiratory syncytial virus, three for parainfluenza, and 3 for adenovirus. Almost all patients received antiviral therapy (105 [98.1%]). Among them, 95 (88.8%) patients received oseltamivir and 33 (30.8%) patients received arbidol. Glucocorticoids were administered in 62 [57.9%] patients. Oxygen therapy was applied in (80 [74.8%] patients. In total, 20 patients required invasive mechanical ventilation. On day 1 of invasive mechanical ventilation, the median PaO_2_/FiO_2_ ratio was 103 (IQR 58–172) and the median APACHE II score was 25 (IQR 17–32). Three patients received extracorporeal membrane oxygenation (ECMO) therapy, Two of them survived and were discharged at day 26 and day 32, and one died due to sudden cardiac arrest after connection to the ECMO circuit. The causes of death included refractory ARDS (15 [78.9%]), septic shock (1 [5.3%]), sudden cardiac arrest (1 [5.3%]), hemorrhagic shock (1 [5.3%]), and acute myocardial infarction (1 [5.3%]).

**Table 3:** Complications and treatment measure of COVID-19 patients

	Total (*n* = 107)	Survivors (*n* = 88)	Non-survivors (*n* = 19)
Complications			
Shock	22 (20.6)	3 (3.4)	19 (100.0)
Acute cardiac injury	12 (11.2)	4 (4.5)	8 (42.1)
ARDS	28 (26.2)	11 (12.5)	17 (89.5)
Acute kidney injury	14 (13.1)	0 (0.0)	14 (73.7)
Evidence of co-infection			
Bacterial	5 (4.7)	1 (1.1)	4 (21.1)
Viral	12 (11.2)	10 (11.4)	2 (10.5)
Treatment			
Antiviral therapy	105 (98.1)	87 (98.9)	18 (94.7)
Oseltamivir	95 (88.8)	77 (87.5)	18 (94.7)
Arbidol	33 (30.8)	33 (37.5)	0 (0.0)
Antibiotic therapy	85 (79.4%)	67 (76.1)	18 (94.7)
Glucocorticoid therapy	62 (57.9)	44 (50.0)	18 (94.7)
CRRT			
Oxygen therapy	4 (3.7)	0 (0.0)	4 (21.1)
Oxygen inhalation	80 (74.8)	78 (88.6)	2 (10.5)
Non-invasive ventilation	7 (6.5)	7 (8.0)	0 (0.0)
IMV alone	17 (15.9)	1 (1.1)	16 (84.2)
IMV plus ECMO	3 (2.8)	2 (2.3)	1 (5.3)

Data are *n* (%). *P* values indicate differences between survivors and non-survivors. *P* < 0.05 was considered significant

*ARDS* acute respiratory distress syndrome, *CRRT* continuous renal replacement therapy, *IMV* invasive mechanical ventilation, *ECMO* extracorporeal membrane oxygenation

### Risk factors associated with death for COVID-19

On the univariate analysis, the risk factors associated with death at hospital admission were older age, male gender, hypertension, diabetes, cardiovascular disease, raised white blood cell counts, elevated level of neutrophil counts, thrombocytopenia, creatine kinase-MB, lactate dehydrogenase, alanine aminotransferase, aspartate aminotransferase, and creatinine (Table 4). On the multivariable analysis, older age and male gender remained the significant independent risk factors for death (Table 5).

**Table 4:** Univariate analysis of variable associated with death for COVID-19 patients

Variable	Univariable	
	OR (95% CI)	*P* value
Age	1.102 (1.054–1.152)	< 0.001*
Male	6.114 (1.662–22.485)	0.006*
Hypertension	5.000 (1.748–14.301)	0.003*
Diabetes	4.881 (1.310–18.184)	0.018*
Cardiovascular disease	7.972 (2.290–27.753)	0.001*
White blood cell count	1.239 (1.055–1.455)	0.009*
Neutrophil count	1.257 (1.073–1.472)	0.005*
Lymphocyte count	0.234 (0.051–1.075)	0.062
Platelet count	0.987 (0.977–0.997)	0.009*
Prothrombin time	1.084 (0.737–1.595)	0.683
Activated partial thromboplastin time	0.998 (0.979–1.017)	0.829
Creatine kinase	1.001 (0.999–1.002)	0.277
Creatine kinase-MB	1.043 (1.008–1.079)	0.015*
Lactate dehydrogenase	1.006 (1.002–1.010)	0.004*
Alanine aminotransferase	1.020 (1.002–1.038)	0.031*
Aspartate aminotransferase	1.034 (1.015–1.054)	< 0.001*
Total bilirubin	1.070 (0.995–1.149)	0.066
Blood urea nitrogen	1.001 (0.985–1.016)	0.943
Creatinine	1.037 (1.015–1.058)	0.001*
Tamiflu	0.389 (0.047–3.209)	0.380

*MB* muscle and brain type

*P < 0.05 was considered significant

**Table 5:** Univariate and multivariate analysis of risk factors associated with death for COVID-19 patients

Variable	Univariable		Multivariable	
	OR (95% CI)	*P* value	OR (95% CI)	*P* value
Age (years)	1.102 (1.054–1.152)	< 0.001*	1.111 (1.042–1.184)	0.001*
Male	6.114 (1.662–22.485)	0.006*	7.224 (1.298–40.190)	0.024*
Hypertension	5.000 (1.748–14.301)	0.003*	1.099 (0.264–4.580)	0.897
Cardiovascular disease	7.972 (2.290–27.753)	0.001*	1.188 (0.182–7.765)	0.857
Creatinine concentration	1.037 (1.015–1.058)	0.001*	1.012 (0.987–1.037)	0.342

*P < 0.05 was considered significant

## Discussion

Studies on COVID-19 have generally been limited to the description of the initial clinical, hematological, radiological, and microbiological findings. Herein, we first described the clinical course of virologically confirmed COVID-19. This study enrolled 107 discharged patients with COVID-19 which included 88 survivors and 19 non-survivors. We also analyzed the prognosis factors and found that age and male gender were the independent risk factor for mortality.

This study showed the clinical course of COVID-19 presented as a tri-phasic pattern. Week 1 was characterized by fever, cough, dyspnea, and other systemic symptoms. Most positive NAAT results could be obtained in week 1, which suggested that the symptoms were largely related to the effect of viral replication. In surviving patients, laboratory abnormalities included lymphopenia and prolonged prothrombin time. In non-survivors, the emergence of systemic inflammation was evidenced by higher fever, respiratory rate, WBC counts, and neutrophil counts. Subsequently, multiple organ dysfunction syndrome (MODS) occurred with thrombocytopenia, renal failure, acute myocardial injury, and ARDS. Notably, there was an obvious drop in body temperature around day 7, probably in relation to the widespread use of methylprednisolone as a rescue therapy.

During weeks 2 of illness, the NAAT test became negative in surviving patients at a median of 13 days after illness onset. At the same time, fever, cough, and systemic symptoms began to resolve. However, lymphocyte counts still remained low, even as symptomatic illness was resolved. This suggests that the lymphocytes are the main target of SARS-CoV-2 infection, and the lymphocyte counts need some time to recover. In the non-survivors, clinical status deteriorated and MODS developed during the second week.

In week 3, the organ functions improved in survivors but continued to deteriorate the non-survivors. The lymphocyte counts dropped further, and immune dysfunction became obvious in the non-survivors. These patients developed severe ARDS necessitating ventilation and even ECMO support, septic shock supported by vasopressors, and an end-stage renal failure requiring continuous renal replacement therapy. Coagulation dysfunction and thrombocytopenia also developed. Death was inevitable due to multi-organ failure.

Notably, most non-survivors in our study were old male. Multivariate analysis showed older age and male gender were independent risk factors for death. A recent study examining single-cell RNA expression profiling of angiotensin-converting enzyme 2 (ACE2), the cellular receptor of SARS-CoV-2, showed that Asian males had an extremely large number of ACE2-expressing cells in the lung [17, 20]. A finding that might underlie the higher risk of death in this population.

After the incubation period, the frequent manifestations of COVID-19 were fever, cough, dyspnea, and bilateral infiltrates on chest imaging [10–12]. Evidence has shown that SARS-CoV-2 was found in the loose stool of a patient, and potential transmission through the fecal-oral route should be considered [18, 19]. Consistent with the finding, some patients showed digestive symptoms (e.g., abdominal pain, diarrhea, nausea, and vomiting) at the illness onset. Multi-lobar involvement on initial chest CT was shown in most of our patients, consistent with a primary pulmonary method of acquisition. Notably, the mean arterial pressure was higher in non-survivors than in survivors because the comorbidity of hypertension was more common in non-survivors.

Until now, no fully proven and specific antiviral treatment for the SARS-CoV-2 infection exists. Organ support therapy is the cornerstone in the treatment of critically ill patients with SARS-CoV-2 infection. Remdesivir, a novel nucleotide analog antiviral drug, has been used in the first case with COVID-19 in the USA, and a clinical trial of remdesivir in SARS-CoV-2 infection is in progress [21]. Remdesivir and chloroquine have been shown to effectively inhibit the SARS-CoV-2 in Vero E6 cells [22]. In hospitalized adult patients with severe COVID-19, no benefit was observed with lopinavir-ritonavir treatment beyond standard care [23]. Moreover, the effects of abidol, oseltamivir, or methylprednisolone in SARS-CoV-2 infection have not been fully evaluated.

This study has several limitations. First, the virus loads were not detected. We cannot determine if the MODS or severity of illness was correlated with the sustained viral load. Secondly, due to the retrospective study, data about the values of creatine kinase, creatine kinase-MB, and lactate dehydrogenase from day 11 to day 17 were missing. The enzyme activity could not be analyzed in week 3 after illness onset. Further study should be conducted to clarify the dynamic change of the three lab index. Third, only 107 patients with confirmed SARS-CoV-2 infection were enrolled in this study. Future studies should be needed to enroll larger sample sizes to evaluate the clinical course and analyze the risk factor for death in COVID-19.

## Conclusions

Our experience in Wuhan revealed a period of 7–13 days after the onset of illness as the critical stage in the COVID-19 course. Age and male gender were independent risk factors for death of COVID-19.

## Supplementary information

**Additional file 1: Figure S1**. Dynamic Body Temperature and Laboratory Findings in 107 COVID-19 Patients. Timeline charts illustrate the temperature and laboratory parameters in 107 patients with COVID-19 (88 survivors and 19 non-survivors) every other day based on the days after the onset of illness. The dashed lines in red show the upper normal limit of each parameter, and the dashed line in blue shows the lower normal limit of lymphocyte count. * *P* <0 .05 for survivors vs non-survivors.

## Data Availability

The datasets used and/or analyzed during the current study are available from the corresponding authors on reasonable request.

## Abbreviations

COVID-19: Coronavirus disease 2019
SARS-CoV-2: Severe acute respiratory syndrome coronavirus 2
MERS: Middle East respiratory syndrome
AKI: Acute kidney injury
ARDS: Acute respiratory dyspnea syndrome
IQR: Interquartile range
NAAT: Nucleic acid amplification test
PT: Prothrombin time
APACHE II score: Acute Physiology and Chronic Health Evaluation II score
ECMO: Extracorporeal membrane oxygenation
MODS: Multiple organ dysfunction syndrome
ACE2: Angiotensin-converting enzyme 2
