# Supplementary figures and images for: Clinical course and outcome of 107 patients infected with the novel coronavirus, SARS-CoV-2, discharged from two hospitals in Wuhan, China
Source: Crit Care. 2020 Apr 30;24:188. doi: 10.1186/s13054-020-02895-6 (PMC7192564; doi:10.1186/s13054-020-02895-6)

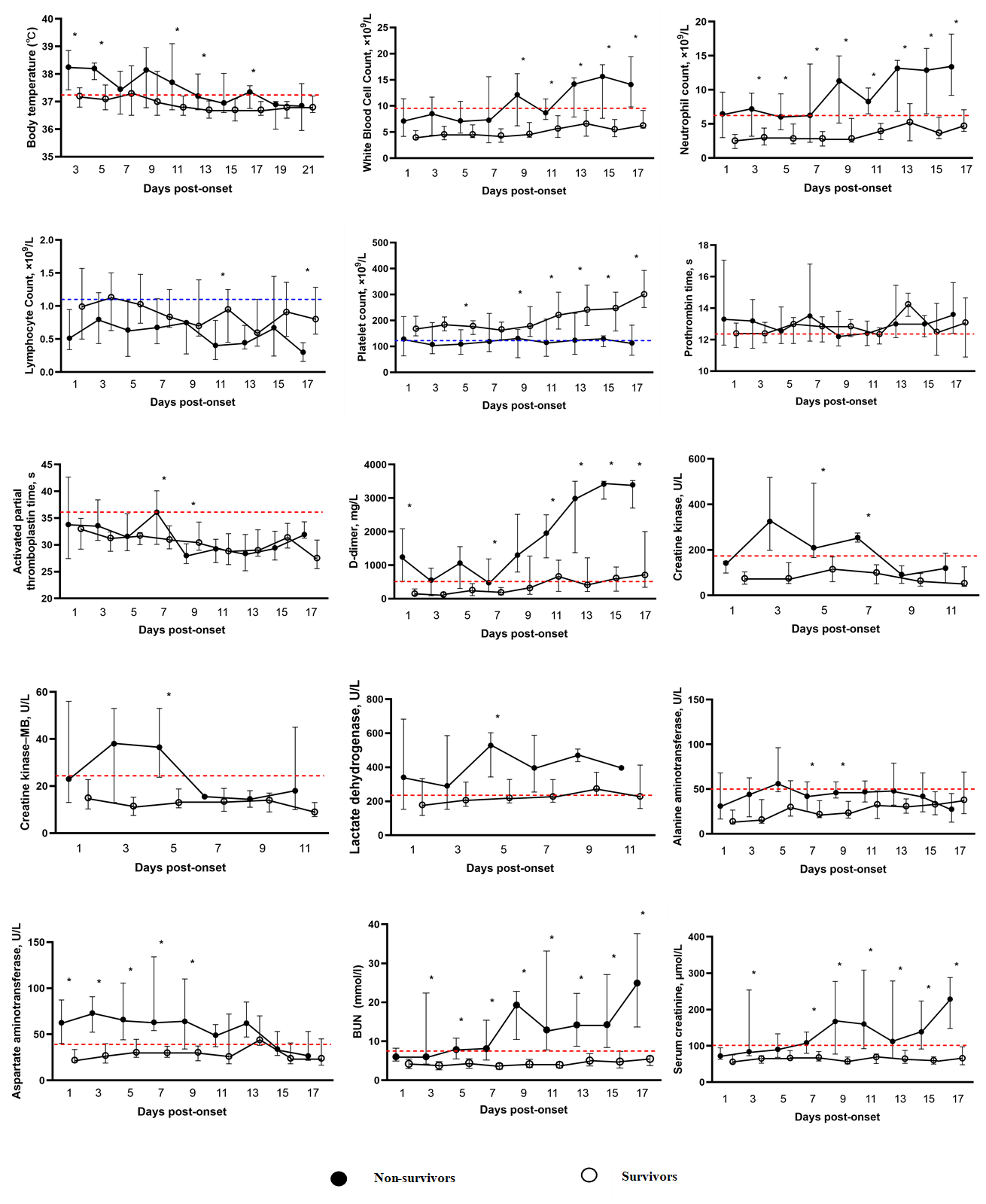

Supplement: Supplementary file 1 — Additional file 1: Figure S1. Dynamic Body Temperature and Laboratory Findings in 107 COVID-19 Patients. Timeline charts illustrate the temperature and laboratory parameters in 107 patients with COVID-19 (88 survivors and 19 non-survivors) every other day based on the days after the onset of illness. The dashed lines in red show the upper normal limit of each parameter, and the dashed line in blue shows the lower normal limit of lymphocyte count. * P <0 .05 for survivors vs non-survivors. [file 13054_2020_2895_MOESM1_ESM.docx]
